# Etymologia: Coxsackievirus

**DOI:** 10.3201/eid1811.ET1811

**Published:** 2012-11

**Keywords:** etymologia, coxsackievirus, Coxsackie, New York, Dalldorf, suckling mice

Named for Coxsackie, the small town on the Hudson River where they were first isolated, human coxsackieviruses are nonenveloped, positive-sense, single-stranded RNA viruses in the family *Picornaviridae*, genus *Enterovirus.* They were first described by Gilbert Dalldorf, who was investigating suspected poliomyelitis outbreaks in upstate New York in the summer of 1947. Coxsackieviruses are divided into 2 groups, A and B. In suckling mice, group A viruses cause generalized myositis and flaccid paralysis, and group B viruses cause focal myositis and spastic paralysis. With the discovery of coxsackieviruses, Dalldorf also helped popularize the suckling mouse as an inexpensive laboratory animal model.

## References

[1] Dalldorf G. The Coxsackie viruses. Bull N Y Acad Med. 1950;26:329–35. 15411582 PMC1929947

[2] Dalldorf G, Sickles GM, Plager H. Gifford R. A virus recovered from the feces of “poliomyelitis” patients pathogenic for suckling mice. J Exp Med. 1949;89:567–82. 10.1084/jem.89.6.567 18144319 PMC2135891

[3] Dorland’s Illustrated Medical Dictionary. 32nd ed. Philadelphia: Elsevier Saunders; 2012.

[4] Racaniello V. Coxsackie NY and the virus named after it, August 10, 2009 [cited 2012 Aug 21]. http://www.virology.ws/2009/08/10/coxsackie-ny-and-the-virus-named-after-it/

[5] Tao Z, Song Y, Li Y, Liu Y, Jiang P, Lin X, Coxsackievirus B3, Shandong Province, China, 1990–2010. Emerg Infect Dis. 2012;18:1865–7. 10.3201/eid1811.120090 23092737 PMC3559141

